# Schnitzler Syndrome: A Recherche Entity

**DOI:** 10.7759/cureus.13338

**Published:** 2021-02-14

**Authors:** Rajesh Kumar, Anupama Behera, Ratul Seal, Subhabrata Patra

**Affiliations:** 1 Internal Medicine, All India Institute of Medical Sciences, Bhubaneswar, IND

**Keywords:** monoclonal igm gammopathy, mpgn, puo, schnitzler syndrome, rash

## Abstract

Schnitzler syndrome (SS) is a rare disease of unknown etiology. Literature suggests that only around 300 well-diagnosed cases have only been reported worldwide and rarely from India. This syndrome has a slight male predominance with a mean age of onset of around 50 years. It is considered an autoinflammatory disease with presentation mimicking adult-onset Still's disease and systemic lupus erythematosus, and its presentation most commonly includes recurrent fever, urticarial rash, arthralgia, and bone pains. The probable pathogenesis is considered to be cytokine-mediated, mostly interleukin- 1 (IL-1), and its association with the NRLP3 gene has been mentioned in a few reports.

Herein, we report a case of a 40-year-old female who presented to us with fever, jaundice, rash, and pedal edema, and detailed investigations revealed leukocytosis with low complements, normal bone marrow with an 'M band' in the immunoglobulin M (IgM) region. Skin biopsy was suggestive of leukocytoclastic vasculitis and renal biopsy was suggestive of membranoproliferative glomerulonephritis (MPGN). All autoimmune and viral markers were negative, including cryoglobulins, and by excluding all possible differentials, the diagnosis of Schnitzler syndrome was confirmed.

SS is a disease of exclusion and several autoimmune, hematological infections need to be excluded, hence, this requires extensive workup. It’s the rarest of rare cases, with a variable presentation, specially pyrexia of unknown origin (PUO) with rash, hence this case will open the physician's vision of undiagnosed cases, and further research will help understand its pathogenesis.

## Introduction

Schnitzler syndrome is a rare, orphan, acquired systemic disease simulating auto-inflammatory syndromes. It was first reported in 1972 and subsequently published in 1974 by Liliane Schnitzler, a French dermatologist [1].

Schnitzler syndrome usually presents with a history of recurrent fever, urticarial skin rash off and on, arthralgia with other various presentations as in anti-inflammatory disease. Monoclonal immunoglobulin (IgM) in serum is the cornerstone finding with a rare variant of IgG. Diagnostic criteria include Lipsker's and Strasbourg criteria [2]. The probable pathogenesis is interleukin-1 (IL-1) mediated and is an acquired disease involving the abnormal stimulation of the innate immune system.

The lymphoproliferative disorder develops in about 15% to 20% of patients with a Schnitzler’s syndrome sharing [3]. AA amyloidosis occurs in untreated patients of SS [4]. Conventional therapies, including anti-histamines for the skin rash, as well as anti-inflammatory drugs and immunosuppressive drugs for the systemic signs, are usually ineffective. However, the IL-1 receptor antagonist anakinra was found to rapidly control all the symptoms of this syndrome. Biologics like canakinumab and rilonacept are new drugs.

## Case presentation

A 40-year-old female was admitted with 15 days of fever, jaundice, and joint pains with no swelling, predominately in the small joints of the hand. The patient had a history of four hospital admissions outside, in the last six months, for recurrent low-to-moderate grade intermittent fever with arthralgia for which antibiotics were given. However, the diagnosis could not be established even after a detailed workup. 

Initial investigations revealed anemia, polymorpho-leukocytosis (Hb%: 8 gm%, total leucocyte count: 24000/mm^3^, total platelet count: 4.32 lac/mm^3^), renal function test was in the normal range, liver function revealed conjugated hyperbilirubinemia with transaminitis (total serum bilirubin: 3.6 mg/dl, direct bilirubin: 2.0 mg/dl, aspartate aminotransferase (AST): 700 IU/ml, alanine transaminase (ALT) 800 IU/ml). Viral serology markers, including human immunodeficiency virus (HIV), hepatitis A, B, C, and E, Cytomegalovirus, Epstein-Barr virus, and parvovirus B19 were negative. Autoimmune markers, including vasculitis workup (anti-LKM 1, 2, and 3, SMA, AMA, ANA, ANA profile & Anti-ds-DNA titer, cANCA, pANCA, RA factor, anti-CCP) were within the normal range. Infectious workup was normal (sterile cultures of blood and urine, normal serum procalcitonin). Anemia workup was suggestive of anemia of chronic disease (direct Coombs test (DCT) negative, lactate dehydrogenase (LDH) normal, serum ferritin mildly raised, corrected reticulocyte count normal).

**Figure 1:**
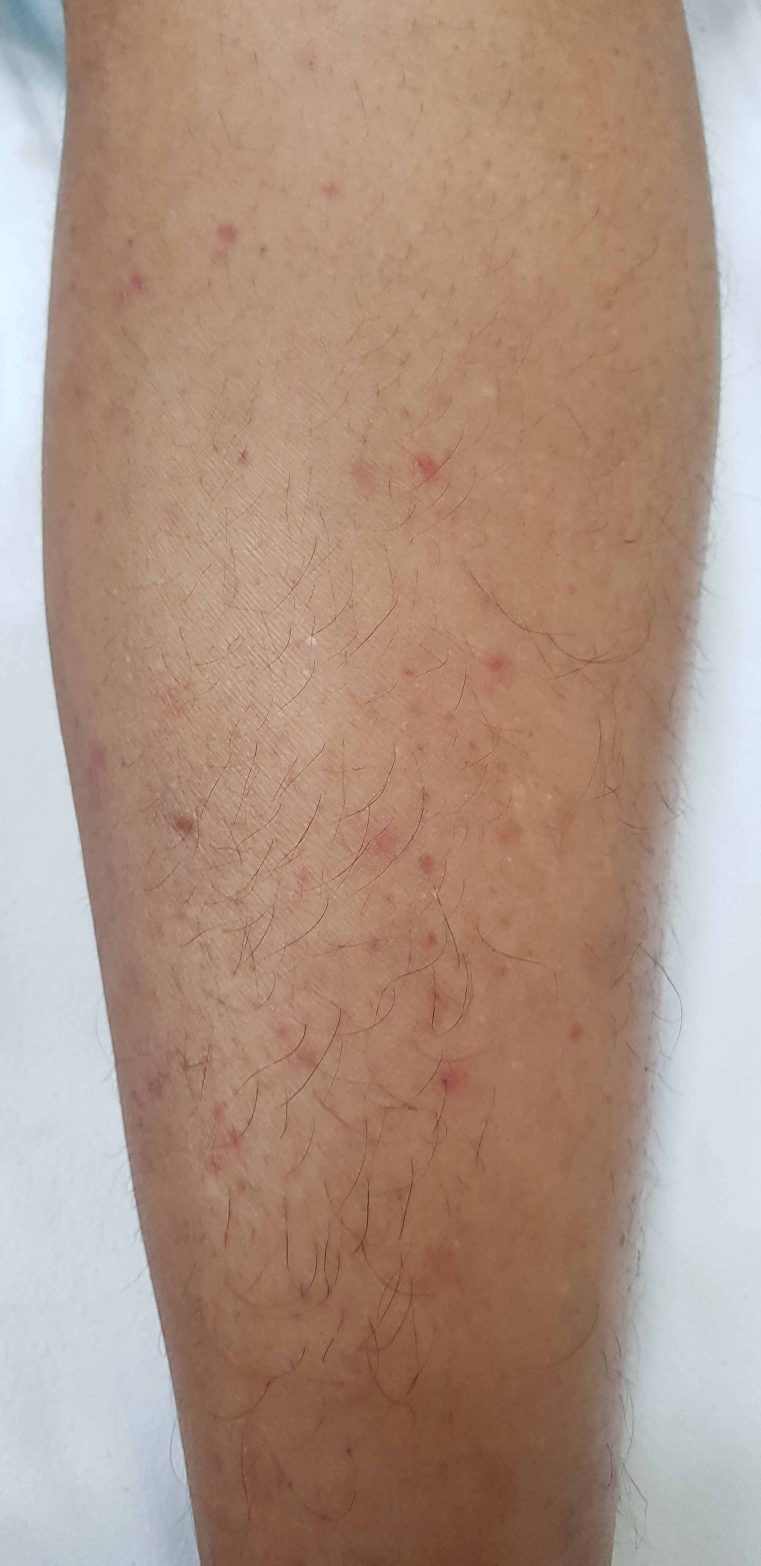
Maculopapular, red-colored, mild, pruritic rash on lower limb

Gradually, jaundice subsided but fever persisted with leukocytosis, and she started developing lower limb swelling with new onset of maculopapular, mild, itchy eruptions on the abdomen and upper limbs with subsequent spreading to the whole body (Figure 1). Skin biopsy was done and was suggestive of leukocytoclastic vasculitis. Serum cryoglobulins and Congo Red staining were negative. Serum complements (C3 and C4) were low. Twenty-four-hour urinary protein was increased (1 gm) and renal biopsy was suggestive of type 1 membranoproliferative glomerulonephritis (MPGN). Bone marrow was hypercellular, serum protein electrophoresis was suggestive of monoclonal gammopathy in the IgM and kappa regions, with increased free kappa light chain values and normal kappa lambda ratio (Sr kappa-lc: 344mg/l (3.3-19.4), IgM: 627 mg/dl (40-230), free kappa/lambda ratio: 16). Urine electrophoresis revealed kappa lc: 393, and the Bence Jones protein was absent. Abdominal sonography and computed topographies (CTs) of the thorax and abdomen revealed mild hepatomegaly. MRI spine, bone scan, and 2D echocardiography were normal.

With all these findings, Schnitzler syndrome was diagnosed as per Strasbourg diagnostic criteria after ruling out all possible differentials. The patient did not respond to broad-spectrum antibiotics given during her stay of 20 days but responded to pulse methylprednisolone subsequently given after the diagnosis. Anakinra (IL-1 antagonist) was planned but could not be given because of financial problems. The patient is in periodic follow-up for two years (since 2017) and is currently on low-dose steroids and colchicine (during acute flares of skin rash). She is doing well with two to three flares till now, with no proteinuria, fever, or arthralgia.

## Discussion

This autoinflammatory syndrome clinically presents with a diverse constellation of symptoms, making its initial diagnosis very challenging [5]. In this patient, from the physician's perspective, it was challenging and mesmerizing, as various presentations unfolded gradually, and the possibilities of multiple myeloma, amyloidosis, and IgM monoclonal gammopathies were always on the cards. However, there were findings that could not be explained from older case reports like jaundice and MPGN though there was a case reported about MPGN in the same year [6]. 

The pathophysiology of the fever and of the syndrome, in general, remains unclear. Previous studies showed a disturbed cytokine balance. The presence of anti-IL-1 antibodies was reported with increased frequency in this syndrome by Saurat et al. [7], but this finding was subsequently not confirmed by other investigators. Previous studies suggested that IgM deposits in the dermo-epidermal junction in the skin involved in the pathophysiology of the rash is of the same isotope as that of IgM present in monoclonal gammopathy of SS [8].

The main unresolved question is whether the clonal IgM proliferation is primitive in nature or the result of continuous antigenic stimulation. Thus the question of whether the Schnitzler syndrome is a smoldering lymphoproliferative disorder with systemic expression, comparable to the POEMS (polyneuropathy, organomegaly, endocrinopathy, monoclonal gammopathy, and skin changes) syndrome or a systemic disorder with an accompanying IgM, remains unanswered. In rare instances, the treatment of the underlying lymphoproliferative disorder had a beneficial effect on Schnitzler syndrome, supporting the former point of view [9].

## Conclusions

SS is a rare disease with myriad presentations as in our case than usual listed in the literature. The diagnosis is essentially that of exclusion with limited experience of long-term outcomes of the disease. Autoimmune diseases like systemic lupus erythematosus (SLE), vasculitis, adult-onset Still’s disease, and pyrexia of unknown disease are common mimics. Treatment remains challenging, especially in resource-limited settings. As we see herein, the patient is doing very well with steroids, not requiring immunosuppressants till reported.
